# Proneness to infections and familial risk of tic disorders

**DOI:** 10.1017/S003329172610419X

**Published:** 2026-04-22

**Authors:** Josep Pol-Fuster, Sara Vasiljevic, Lorena Fernández de la Cruz, Jan C. Beucke, Eva Hesselmark, James J. Crowley, Isabell Brikell, Elles de Schipper, Brian M. D’Onofrio, Zheng Chang, Henrik Larsson, Kristina Tedroff, Paul Lichtenstein, Ralf Kuja-Halkola, Selma Idring, David Mataix-Cols

**Affiliations:** 1 Centre for Psychiatry Research, Department of Clinical Neuroscience, Karolinska Institutet & Stockholm Health Care Services, Region Stockholm, Stockholm, Sweden; 2 Institute for Systems Medicine, Department of Human Medicine, MSH Medical School Hamburg, Hamburg, Germany; 3 Department of Genetics, University of North Carolina at Chapel Hill, NC, USA; 4 Department of Medical Epidemiology and Biostatistics, Karolinska Institutet, Stockholm, Sweden; 5 Department of Global Public Health and Primary Care, University of Bergen, Bergen, Norway; 6 Department of Biomedicine, Aarhus University, Aarhus, Denmark; 7 Department of Psychological and Brain Sciences, Indiana University, Bloomington, IN, USA; 8 School of Medical Sciences, Örebro University, Örebro, Sweden; 9 Neuropediatric Unit, Department of Women’s and Children’s Health, Karolinska Institutet, Stockholm, Sweden; 10 Department of Clinical Sciences, Lund University, Lund, Sweden

**Keywords:** chronic tic disorder, cohort study, familial co-aggregation study, infections, PANDAS, PANS, Tourette syndrome

## Abstract

**Background:**

Postinfectious autoimmune processes are hypothesized to be causally implicated in tic disorders, including Tourette syndrome and chronic tic disorder. However, this hypothesis remains controversial. In this nationwide cohort study, we aimed to clarify the mechanisms underlying the association between proneness to infections and tic disorders.

**Methods:**

Using Swedish national registers, we identified 3,886,533 individuals (probands) born between 1970 and 2008 with available data on both biological parents. Probands were linked to six clusters of relatives: parents, full siblings, maternal half-siblings, paternal half-siblings, aunts/uncles, and cousins. Cox proportional hazards regression models were used to estimate the risk of tic disorders in probands exposed to infections and their relatives, compared with unexposed probands and their relatives. We also examined dose–response associations using logistic regression models.

**Results:**

Probands exposed to infections had an increased risk of tic disorders (hazard ratio [HR], 1.46; 95% confidence interval [CI], 1.40–1.52), as did their relatives. The observed risks increased with the degree of genetic relatedness, from HR (95% CI) of 1.15 (1.12–1.19) in cousins to 1.31 (1.25–1.37) in first-degree relatives. There was a dose–response association between the number of infections in the probands and the odds for tic disorders in the probands and their relatives. Results remained consistent after adjustment for infections in relatives, tic disorders in probands, and autoimmune diseases in probands and relatives.

**Conclusions:**

Our results suggest an important role of shared genetic factors in the association between infections and tic disorders, potentially pointing to pleiotropic mechanisms.

## Introduction

Tic disorders, including Tourette syndrome and chronic tic disorder, are highly heritable neuropsychiatric conditions characterized by motor and/or vocal tics (Browne et al., 2015; Mataix-Cols et al., 2015). Gene searching efforts are well underway and genome-wide association studies (GWAS) are rapidly approaching the discovery zone (Lin, Tsai, & Chou, 2022; Strom et al., 2025; Tsetsos et al., 2024). Environmental risk factors such as obstetric and perinatal complications (Brander et al., 2019) are also thought to play a role in the etiology of tic disorders.

Postinfectious autoimmune processes, particularly related to group A streptococcal (GAS) infections, have been proposed as potential causal factors for the development of tic disorders (Orlovska et al., 2017; Swedo et al., 1998). However, after more than two decades of research, this hypothesis still lacks solid evidence (Gagliano, Carta, Tanca, & Sotgiu, 2023). Two of the largest studies on the topic conducted to date failed to find significant associations between GAS infections and the onset of tics in at-risk individuals (Schrag et al., 2022) or the exacerbation of tics in children with tic disorders (Martino et al., 2021). A large population-based study of about three million individuals conducted in Sweden found an increased risk of subsequent tic disorder diagnoses following severe prenatal and early childhood bacterial and/or viral infections (Zhang et al., 2023). However, in this study, the association was no longer statistically significant in co-sibling analyses, which effectively adjusted for genetic and environmental factors shared by siblings (Zhang et al., 2023), suggesting that the role of infections may not be directly causal, but rather influenced by familial confounding. As the susceptibility to infections is also influenced by genetic factors (Kwok, Mentzer, & Knight, 2021; Nudel et al., 2019), genetic pleiotropy (Gratten & Visscher, 2016) may provide a possible explanation for the association between infections and tic disorders. That is, rather than infections directly causing tic disorders, shared genetic factors might predispose individuals to both infections and tic disorders (Nudel et al., 2019; Zhang et al., 2023).

This study leveraged a nationwide cohort from Sweden to clarify the nature of the association between infections and tic disorders, and tested predictions of a shared genetic factors hypothesis. Based on the existing literature, we hypothesized that: (1) there would be a significant association between infections diagnosed in inpatient and outpatient specialist services and tic disorders at the population level; (2) the biological relatives of individuals with infections would have higher risks of tic disorders, compared to relatives of individuals without infections; (3) the strength of the associations would increase with the degree of genetic relatedness, independent of infections in the relatives; and (4) there would be a dose–response association between infection proneness in the probands and risk of tic disorders in both the probands themselves and in their biological relatives.

## Methods

### Data sources

We used the unique Swedish personal identification number (Ludvigsson, Otterblad-Olausson, Pettersson, & Ekbom, 2009) to link several health and administrative registers. The Total Population Register (Ludvigsson et al., 2016), which includes demographic information on all Swedish residents, as well as emigration and immigration records from and to Sweden since 1961 and 1969, respectively, was used to identify the study cohort and to obtain migration data. The Cause of Death Register (Brooke et al., 2017), covering dates and causes of all deaths since 1961, was used to obtain information on deaths. The Multi-Generation Register, with information about kinship on each person registered in Sweden at some time after 1960 and on those born from 1932, was used to identify biological kinships (Ekbom, 2011). The National Patient Register (NPR) (Ludvigsson et al., 2011), covering all inpatient hospital admissions since 1969 and outpatient specialist care since 2001, was used to obtain data on clinical diagnoses. The Regional Healthcare Data Warehouse of Region Stockholm (VAL), which registers all primary care visits in Stockholm County (Zarrinkoub et al., 2013), was used to identify infections from primary care settings. We had coverage for infections in VAL from 1997 onwards.

### Study population

The main study population included all individuals born in Sweden between January 1, 1970, and December 31, 2008, with available data on both biological parents. We excluded individuals who emigrated or died before the age of 3 (minimal age for the outcome, see below) or before 1987 (when complete inpatient care data became available in the NPR), as well as those diagnosed with tic disorders prior to 1987.

Individuals within this cohort (probands) were used to identify proband-relative pairs and to construct six sub-cohorts of biological relatives with the aim of examining relatives who differ in their degree of shared genetic and environmental factors, including parents, full siblings, maternal half-siblings, paternal half-siblings, aunts/uncles, and cousins (i.e. individuals whose parents are full siblings). These sub-cohorts of relatives were analyzed separately and then grouped into broader groups of first-degree relatives (including parents and full siblings), second-degree relatives (including maternal and paternal half-siblings and aunts/uncles), and third-degree relatives (cousins).

Unlike the other proband-relative pairs, the parents and aunts/uncles proband-relative pairs could potentially include proband-relative pairs with relatives outside the study cohort. To maintain a consistent birth year range and minimize generational differences, we excluded proband-relative pairs whose relatives were born before 1960 or after 1990. We also excluded pairs from these proband-relative pairs if the relatives had died, emigrated, or were diagnosed with tic disorders before 1987.

Probands in the main cohort and relatives in the proband-relative pairs were followed from birth or from January 1, 1987, whichever occurred later, until the date of a tic disorder diagnosis, emigration, death, or end of the follow-up (December 31, 2020), whichever came first.

Because the main nationwide cohort does not include diagnoses from primary care, we identified a new cohort for the analysis using VAL. This cohort consisted of individuals born between January 1, 1980, and December 31, 2008, who were living in Stockholm County in 1997, or born in Stockholm County in 1997 or afterwards. From this cohort, we identified three sub-cohorts of biological relatives with varying degrees of shared environmental and genetic factors, including full siblings, half-siblings, and cousins. Infections were defined as the first recorded instance of an infection in VAL (see ICD-10 codes in Supplementary Table 1). Probands identified in the main VAL cohort and relatives in the proband-relative pairs were followed from birth or from January 1, 1997, whichever occurred later, until the date of a tic disorder diagnosis, emigration, a change of residency out of Stockholm County, death, or end of the follow-up (December 31, 2020), whichever came first.

### Exposure: **infections**

Infections were defined as the first recorded instance in the NPR of any kind of infection (viral, bacterial, or unknown) diagnosed in inpatient or specialist outpatient settings (see the Swedish International Classification of Diseases [ICD] codes in Supplementary Table 1). In the within-individual analysis, probands were considered unexposed until their first diagnosis of infection and exposed afterwards. Relatives were considered unexposed prior to the proband’s date of first diagnosis of infections and exposed afterwards.

To examine dose–response associations, we also categorized the number of infections in the proband as 1, 2, or 3 or more infections. Consistent with previous work (Pol-Fuster et al., 2025), and to avoid counting multiple hospital contacts for the same infection as different infections (e.g. relapses, repeated visits due to unresolved symptoms or changes in diagnosis), each infection was defined as an inpatient or outpatient contact occurring at least 2 months after the previous infection.

### Outcome: tic disorders

We defined the outcome as the first diagnosis of Tourette syndrome or chronic tic disorder in the NPR recorded from the age of 3, to minimize the risk of diagnostic misclassification. In line with prior research (Brander et al., 2019; Isomura et al., 2022), tic disorder cases were ascertained by using a previous validated algorithm that minimizes the inclusion of individuals with only transient tics (Rück et al., 2015). The Swedish ICD diagnostic codes for tic disorders have excellent validity and reliability (Rück et al., 2015).

### Covariates

Covariates included sex and categorical birth year, extracted from the Total Population Register, and autoimmune diseases, defined as the first recorded diagnosis of any autoimmune disease in the NPR (see ICD codes in Supplementary Table 1). The latter were included as covariates because autoimmune diseases are known to be associated with both proneness to infections (Nielsen, Kragstrup, Deleuran, & Benros, 2016) and tic disorders (Mataix-Cols et al., 2018).

### Statistical analysis

We first examined the within-individual association between infections and tic disorders in the entire cohort using a Cox proportional hazard regression model with attained age as the underlying time scale and infections as a time-varying exposure. This model was adjusted for the proband’s sex and birth year (categorized in 10-year increments). We calculated hazard ratios (HRs) and 95% confidence intervals (CIs).

Next, we investigated the familial co-aggregation of infections and tic disorders by comparing the risk of tic disorders in relatives of probands with infections to that in relatives of unexposed probands. To that end, we fitted a series of Cox proportional hazard regression models with attained age as the time scale and the proband’s infection as a time-varying exposure (Model 1). All models were adjusted for the proband’s and the relative’s sex and birth year.

To determine if familial co-aggregation could be better explained by the direct effect of infections, we additionally adjusted Model 1 for infections in the relatives (Model 2). To account for the possibility that the results were influenced by the co-occurrence of tic disorders in both the proband and relative, we additionally adjusted Model 1 for tic disorders in the proband (Model 3). Additionally, we further adjusted Model 1 for autoimmune diseases in relatives (Model 4) and probands (Model 5).

To validate the robustness of our findings across different methods, we repeated the within-individual analysis and the analyses from Models 1, 2, and 3 using logistic regression models with lifetime exposure and outcomes. Finally, in an additional logistic regression analysis, we examined the possible dose–response association between the number of infections (categorized as 1, 2, or 3 or more infections) in the probands (a proxy of infection proneness) and their odds of tic disorders, as well as the odds of tic disorders in their relatives.

Robust standard errors were applied in all analyses to account for familial clustering. Data were analyzed from August 15, 2024, to March 1, 2025. Statistical analyses were conducted in SAS software (version 9.4; SAS Institute) and R, using survival (Therneau, 2023) and drgee packages (Zetterqvist & Sjölander, 2015).

## Results

### Cohort characteristics

The characteristics of the main study cohort are summarized in Table 1. The cohort included a total of 3,886,533 individuals, 1,502,255 (38.7%) of whom had at least one record of infection during the study period. Of the total cohort, 10,143 individuals (0.3%) were diagnosed with either Tourette syndrome or chronic tic disorder (77.9% males). The median age at the time of first tic disorder diagnosis was 12.5 years (interquartile range [IQR]: 7.1), and the median time from the first recorded infection to tic disorder diagnosis was 8.8 years (IQR: 6.0).

**Table 1.** Distribution of study cohort characteristics

		Infections (*N* = 1,502,255) *n (%)*	No infections (*N* = 2,384,278) *n (%)*
Tic disorders	No record of tic disorders	1,497,443 (99.7)	2,378,947 (99.8)
	Tic disorders	4,812 (0.3)	5,331 (0.2)
Sex	Male	661,516 (44.0)	1,335,238 (56.0)
	Female	840,739 (56.0)	1,049,040 (44.0)
Birth year	1970–1979	323,852 (21.6)	663,318 (27.8)
	1980–1989	379,046 (25.2)	596,442 (25.0)
	1990–1999	410,142 (27.3)	628,610 (26.4)
	2000–2008	389,215 (25.9)	495,908 (20.8)

### Familial co-aggregation

**Figure 1.**
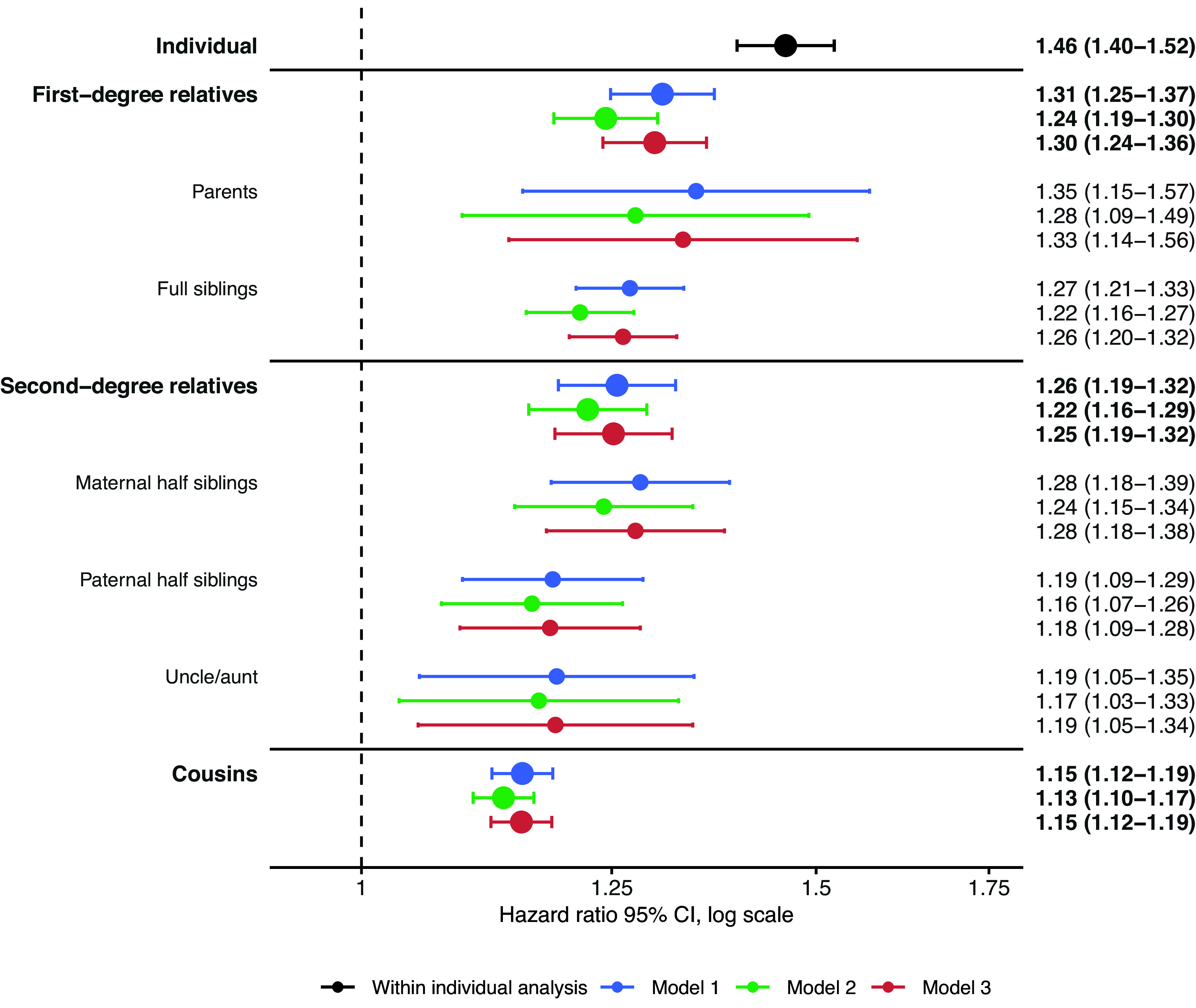
Hazard ratio for tic disorders in relatives of probands diagnosed with infections. *Note:* The within-individual analysis (black) was adjusted for sex and birth year (categorized in 10-year increments). Model 1 (blue) examines the risk of tic disorders for relatives of probands with infections, adjusted for the proband’s and relative’s sex and birth year (categorized in 10-year increments). Model 2 (green) is based on Model 1, additionally adjusting for infections in the relatives. Model 3 (red) is based on Model 1, additionally adjusting for tic disorders in the probands. Abbreviations*:* CI, ‘confidence interval’.

Table 2 presents the total number of individuals included in the analysis for each cluster of relatives. Figure 1 illustrates the within-individual associations as well as the familial co-aggregation patterns between infections and tic disorders. Individuals who were exposed to infections had a higher risk of developing tic disorders, compared to unexposed individuals (HR, 1.46; 95% CI, 1.40–1.52; Figure 1).

**Table 2.** Number of probands in the study cohort and each cluster of relatives

Total cohort and family clusters	Unique probands	Unique pairs^a^	Observations^b^	Excluded
Total cohort	3,886,533	NA	3,886,533	NA
Parents	2,096,775	3,721,912	3,721,912	1,789,758^c^
Full siblings	2,907,686	2,216,405	4,432,810	978,847^d^
Maternal half-siblings	528,881	407,031	814,062	3,357,652^d^
Paternal half-siblings	571,509	470,860	941,720	3,315,024^d^
Aunt/uncle	2,174,317	4,911,583	4,911,583	1,712,216^e^
Cousins	3,062,961	8,570,111	17,140,222	823,572^f^

*Note:* In all pairs of siblings and cousins, each individual contributed to the analysis, at least once, with information on exposure and on outcome.

a Number of the unique pairs identified (e.g. Offspring–Mother, Sibling1–Sibling2).

b Number of observations included in the analysis (i.e. all possible combinations of pairs in which members contribute to the analysis with information on exposure and outcome).

c Probands whose parents were born before 1960 or after 1990, or died, emigrated, or were diagnosed with tic disorders before 1987.

d Probands with no siblings of a certain degree of relatedness identified from the study cohort.

e Probands whose aunts/uncles were born before 1960 or after 1990, aunts/uncles were twins of their parents, or died, emigrated, or were diagnosed with tic disorders before 1987.

f Probands with no cousins identified from the study cohort, double cousins, or if parents of cousins are twins.

Relatives of individuals exposed to infections also exhibited a higher risk of tic disorders, compared to relatives of unexposed individuals. This risk increased with the degree of genetic relatedness. The highest risk was observed in first-degree relatives (HR, 1.31; 95% CI, 1.25–1.37), including parents (HR, 1.35; 95% CI, 1.15–1.57) and full siblings (HR, 1.27; 95% CI, 1.21–1.33). Comparatively, second-degree relatives (HR, 1.26; 95% CI, 1.19–1.32), such as maternal half-siblings (HR, 1.28; 95% CI, 1.18–1.39), paternal half-siblings (HR, 1.19; 95% CI, 1.09–1.29), and aunts/uncles (HR, 1.19; 95% CI, 1.05–1.35), and third-degree relatives, like cousins (HR, 1.15; 95% CI, 1.12–1.19), had progressively lower risks, although confidence intervals overlapped across most clusters of relatives (Model 1 in Figure 1).

The co-aggregation patterns remained similar after adjusting for infections in the relatives, with a slight attenuation of the estimates (Model 2 in Figure 1). Similarly, no significant changes were observed after adjusting for tic disorders in the probands (Model 3 in Figure 1) or when accounting for comorbid autoimmune diseases in both the probands and their relatives (Models 4 and 5 in Supplementary Figure 1). The complementary logistic regression analyses consistently revealed a similar pattern of familial co-aggregation (Models 1, 2, and 3 in Supplementary Table 2). An additional post hoc analysis by infection type revealed a similar familial co-aggregation pattern for bacterial (Supplementary Figure 2) and viral (Supplementary Figure 3) infections.

### Dose–response associations

**Figure 2.**
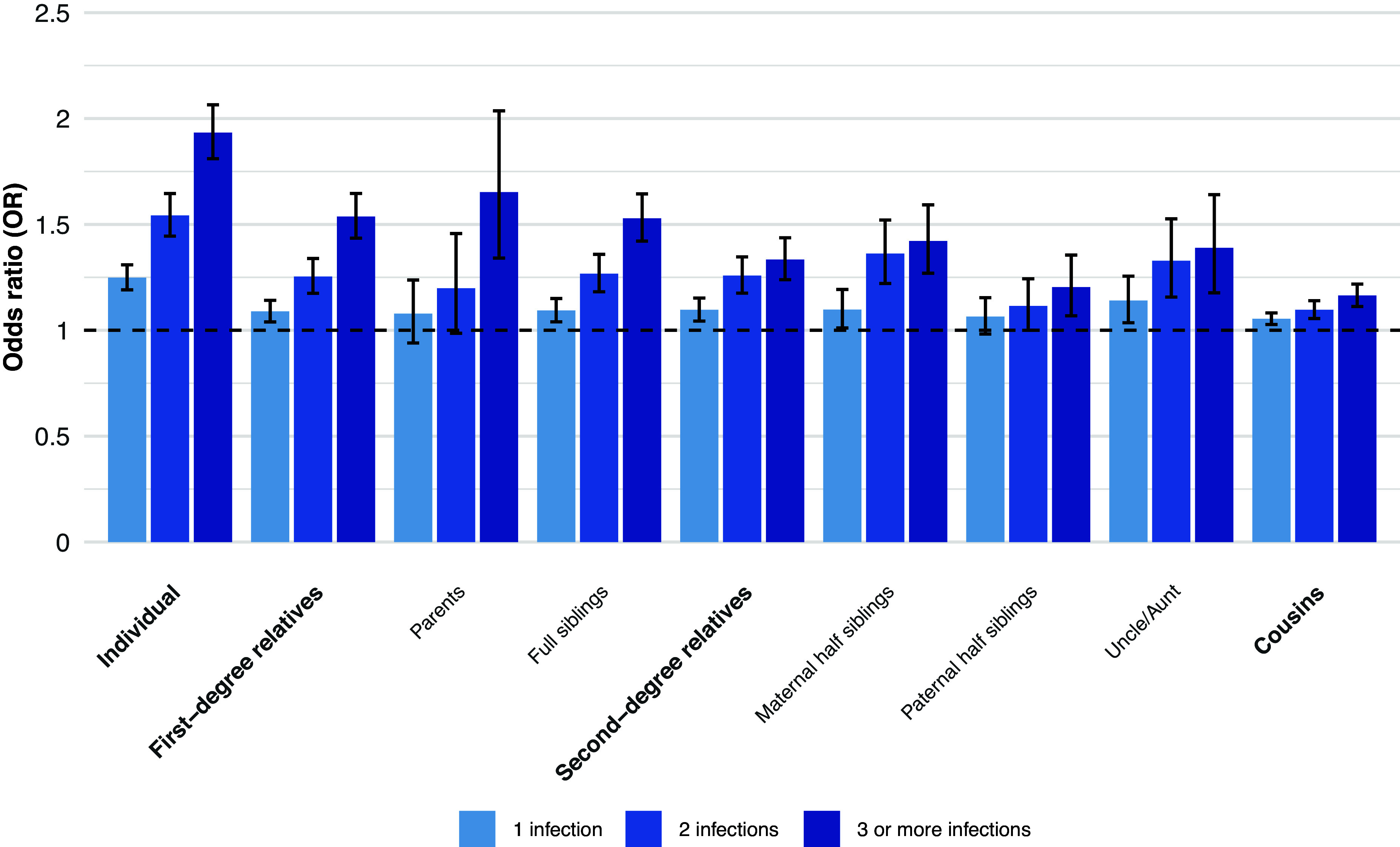
Odds ratio of tic disorders in relatives of probands diagnosed with 1, 2 or 3 or more infections. *Note:* The within-individual analysis was adjusted for sex and birth year (categorized in 10-year increments). The analyses in the relatives were adjusted for the proband’s and relative’s sex and birth year (categorized in 10-year increments).

We observed a dose–response association between the number of infections in the proband and the odds of tic disorder diagnosis. Compared to individuals with no history of infection, the odds of tic disorder diagnosis increased by 25% (OR, 1.25; 95% CI 1.19–1.31) for those with one record of infection, by 54% (OR, 1.54; 95% CI 1.44–1.65) for those with two infections, and by 93% (OR, 1.93; 95% CI 1.81–2.06) for those with three or more records of infections. The odds of tic disorders also increased in relatives according to the number of infections in the proband, with the highest odds of tic disorders observed in the relatives of probands with three or more infections (Figure 2 and Supplementary Table 3).

### Infections registered in primary care

The characteristics of the cohort used for these analyses and the identified clusters of relatives are summarized in Supplementary Table 4 and Supplementary Table 5, respectively. The within-individual association between primary care infections and tic disorders (HR, 1.46; 95% CI, 1.28–1.67; Supplementary Figure 4) was of a similar magnitude as for the infections identified from inpatient and specialist outpatient settings. Additionally, a similar coaggregation pattern was also observed in the different clusters of relatives identified, ranging from 1.33 (95% CI, 1.16–1.54) in full siblings to 1.16 (95% CI, 1.05–1.27) in cousins (Supplementary Figure 4).

## Discussion

To the best of our knowledge, this study is the first to show that infections and tic disorders co-aggregate within families. These results persisted after adjusting for infections among relatives, tic disorders in the probands, and comorbid autoimmune diseases in both the probands and the relatives. Additionally, our results were robust across various analytical approaches and across healthcare settings (specialist vs. primary care). The latter is important because most infections are diagnosed in primary care settings rather than specialist care.

The observed risk patterns in the relatives suggest an important role of shared genetic risk factors in the association between infections and tic disorders. All relatives of probands exposed to infections had an elevated risk of tic disorders, which increased alongside with the degree of genetic relatedness; cousins showed the lowest risk (HR, 1.15; 95% CI, 1.12–1.19), and first-degree relatives the highest (HR, 1.31; 95% CI, 1.25–1.37). Further, the risks in maternal and paternal half-siblings, who both share around 25% of genetic variance with the probands but different degrees of shared environment, were of similar magnitude. In addition, aunts/uncles and cousins, who share approximately 25% and 12.5% of genetic variance with the probands, respectively, but do not share environment, also had increased risks.

We also observed a clear dose–response association between the number of infections in the proband and the risk of tic disorders in both the proband and their biological relatives. The biological relatives of probands with 3 or more infections had the highest risk of developing tic disorders. This provides additional support for the idea that proneness to infections and tic disorders shares genetic risk factors. Finally, our analyses adjusting for infections in relatives suggest that, rather than infections playing a causal role mediated by genetic factors, a pleiotropic mechanism seems a more plausible explanation.

Our findings align well with previous literature suggesting a common genetic susceptibility to infections and psychiatric disorders in general (Nudel et al., 2019), and tic disorders in particular (Zhang et al., 2023). Similar findings were recently reported in obsessive-compulsive disorder (OCD), a disorder that frequently co-occurs with tic disorders and is also hypothesized to be associated with postinfectious autoimmune responses (Pol-Fuster et al., 2025). Interestingly, some small genetic studies have shown associations between genetic variants located in immune-related genes and tic disorders (Keszler et al., 2014). Future tic disorder GWAS studies may provide further insights into this hypothesis by incorporating genetic correlations for susceptibility to infections and tic disorders; to our knowledge, this has not been done previously.

### Strengths and limitations

Strengths include the large population-based cohort using nationwide longitudinal data from the Swedish registries across relatives with different degrees of relatedness, minimizing selection and recall bias. Furthermore, ICD codes for tic disorders in the Swedish NPR are highly valid and reliable (Rück et al., 2015).

Several limitations should also be considered when interpreting the study findings. First, statistical power was generally limited across different clusters of relatives, particularly parents and aunts/uncles, resulting in overlapping CIs. While combining first- and second-degree relatives helped mitigate this issue, overlap in CIs persisted between first-degree relatives and second-degree relatives. However, the observed coaggregation pattern and the consistency across different analytical approaches strengthen the validity of our overall interpretations. Second, our results are based on patients diagnosed in specialist services, meaning that individuals with less severe tic disorders diagnosed by general practitioners may be less well covered. Furthermore, several known factors may influence the likelihood of being diagnosed with tic disorders, including sex, age, ethnicity, and temporal changes in diagnostic practices. These factors were accounted for where possible, although some residual confounding cannot be ruled out. Ethnicity data are not available in the Swedish registers. Finally, our study data are limited to infections resulting in healthcare contact and should therefore be considered a proxy for infection susceptibility rather than a direct measure of biological susceptibility per se. As such, they may be influenced, to some extent, by other factors, such as healthcare-seeking behavior and illness severity. Reassuringly, analyses restricted to infections identified in primary care showed a similar pattern of results.

## Conclusion

Our results suggest an important role of shared genetic factors in the association between infection proneness and tic disorders.

## Supporting information

10.1017/S003329172610419X.sm001 Pol-Fuster et al. supplementary material Pol-Fuster et al. supplementary material

## Data Availability

Sharing of the individual-level data is restricted by Swedish data protection laws and data underlying the reported findings cannot be deposited in publicly accessible archives. In this study, data were obtained from population registers held by the Swedish National Board of Health and Welfare (Socialstyrelsen; http://www.socialstyrelsen.se/english) and Statistics Sweden (SCB; http://www.scb.se/en/). For further information or enquiries about access to the data, any interested parties can contact the data owners, Socialstyrelsen via registerservice@socialstyrelsen.se and SCB via information@scb.se.

## Supplementary Material

The supplementary material for this article can be found at http://doi.org/10.1017/S003329172610419X.
