# Supplementary material for: Proneness to infections and familial risk of tic disorders
Source: Psychol Med. 2026 Apr 22;56:e108. doi: 10.1017/S003329172610419X (PMC13112282; doi:10.1017/S003329172610419X)
Supplement: Pol-Fuster et al. supplementary material [file S003329172610419Xsup001.docx]

**SUPPLEMENTARY MATERIAL**

**Supplementary Table 1: List of Swedish International Classification of Diseases (ICD) codes to collect records of infections and autoimmune diseases from the National Patient Register.**

| **Swedish ICD codes ^a^** | **Viral infections** | **Bacterial infections** | **Unknown microorganism (viral or bacterial)** | **Any autoimmune disease** |
| --- | --- | --- | --- | --- |
| **ICD-8^b^** |  |  |  | 0341 13607 24200 24503 25810 26910 28700 28710 340 35401 390 391 392 44609 44630 44638 44640 56300 56310 57190 580 582 694 69610 69619 69620 69621 69622 69623 70400 71200 71210 71239 71600 73300 73400 73410 |
| **ICD-9** | 008H 008J 008K 008L 008M 008W 045 046 047 048 049 05 06 070 071 072 074 075 077B 077C 077D 077E 077W 077X 078A 078B 078E 078F 078G 078H 078W 079 321E 321H 480 487 711F 790W | 001 002 003 004 005 008A 008B 008C 008D 008E 008F 01 02 030 031 032 033 034B 035 036 037 038 039 041 073 076 077A 078D 078J 080 081 082 083 087 091 092 093 094 095 096 097 098 099A 099B 099C 100 101 102 103 104 320 324 325 326 382 390 391 475 481 482 510 513 540 541 542 590 595 597A 599A 614 615 616 646F 646G 68 711A 711E 790H | 466A 466B 478B 478C | 034B 136B 242A 245C 258B 287A 287D 340 357A 358A 390 391 392 446A 446B 446F 446G 555 556 571F 579A 580 582 694A 694E 694F 696 704A 710A 710B 710C 710D 710W 714A 725 |
| **ICD-10** | A08 A8 A9 B0 B1 B2 B30 B33 B34 B97 G020 G051 H671 J10 J11 J12 J171 J203 J204 J205 J206 J207 J210 M014 M015 | A00 A01 A02 A03 A04 A05 A1 A2 A30 A31 A32 A34 A35 A36 A37 A38 A39 A4 A51 A52 A53 A54 A55 A56 A57 A58 A65 A66 A67 A68 A69 A7 B95 B96 G00 G01 G042 G050 G06 G07 G08 G09 H66 H670 I00 I01 J13 J14 J15 J170 J200 J201 J202 J36 J390 J391 J85 J86 K35 K36 K37 L0 M00 M010 M011 M012 M013 N10 N11 N12 N30 N340 N390 N7 O23 | J200 J201 J202 J390 J391 | A389 D686 D690 D693 E050 E063 E310 G04 G131 G35 G610 G700 I00 I01 I02 L100 L120 L13 L40 L63 K900 K50 K51 K743 M06 M300 M301 M303 M311 M315 M317 M32 M339 M34 M350 M351 M352 M353 N00 N01 N03 N05 |
| ^a^ ICD-8 (1969–1986), ICD-9 (1987–1996), and ICD-10 (1997–onwards)  ^b^ ICD-8 codes were used for autoimmune diseases, individuals may enter the cohort in 1987 with a previous diagnosis of autoimmune diseases. | | | | |

**Supplementary Figure 1: Hazard ratio for tic disorders in relatives of probands diagnosed with infections, adjusted for autoimmune diseases in the probands and in the relatives.**


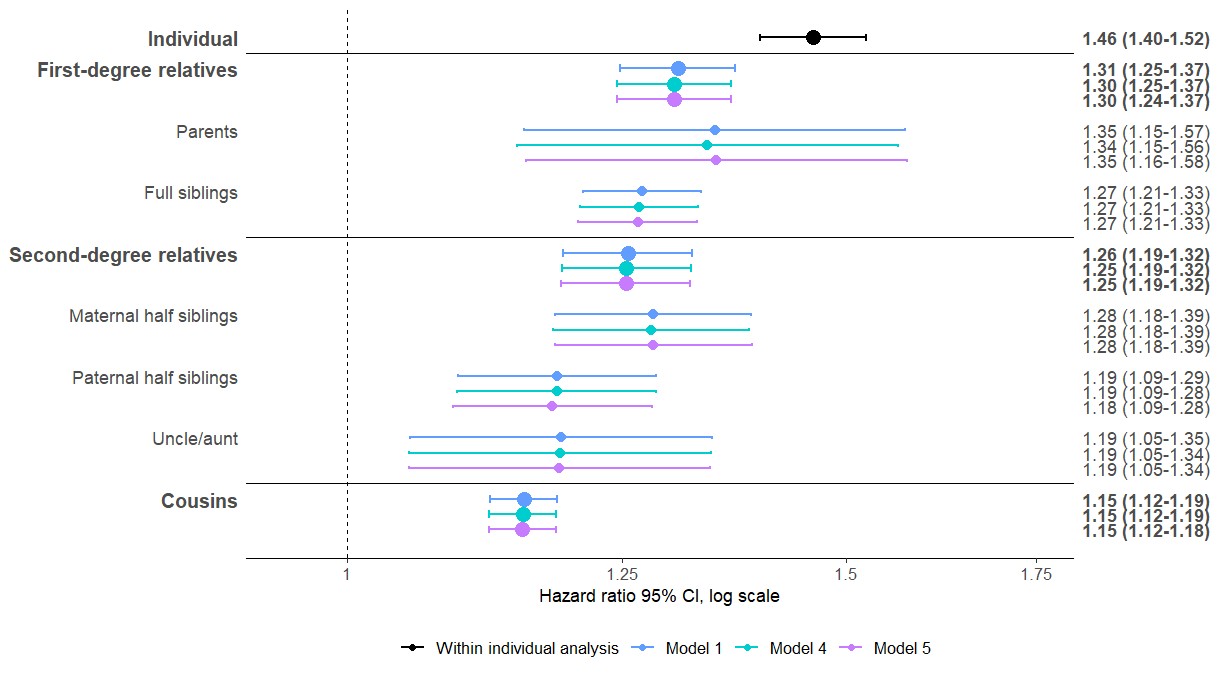


**Footnote:**

The within-individual analysis (black) was adjusted for sex and birth year (categorized in 10-year increments). Model 1 (blue) examines the risk of tic disorders for relatives of probands with infections, adjusted for the proband’s and relative’s sex and birth year (categorized in 10-year increments).

Model 4 (cyan) is based on Model 1, additionally adjusting for autoimmune diseases in the relatives.

Model 5 (purple) is based on Model 1, additionally adjusting for autoimmune diseases in the probands.

*Abbreviations:* CI, confidence intervals.

**Supplementary Table 2: Proportion of tic disorders in relatives of probands diagnosed with infections vs. in relatives of probands with no infections.**

|  | **Relatives of infection probands** | | **Relatives of probands with no infections** | | **Model 1^a^** | **Model 2^b^** | **Model 3^c^** |
| --- | --- | --- | --- | --- | --- | --- | --- |
|  | Total, no. | Tic disorders, no. (%) | Total, no. | Tic disorders, no. (%) | OR (95% CI) | OR (95% CI) | OR (95% CI) |
| First-degree relatives | 3,244,670 | 4,985 (0.15) | 4,910,052 | 6,170 (0.13) | **1.20 (1.16-1.25)** | **1.16 (1.11-1.21)** | **1.19 (1.14-1.24)** |
| Parents | 1,559,291 | 576 (0.04) | 2,162,621 | 645 (0.03) | **1.21 (1.07-1.37)** | **1.16 (1.02-1.31)** | **1.19 (1.05-1.35)** |
| Full siblings | 1,685,379 | 4,409 (0.26) | 2,747,431 | 5,525 (0.20) | **1.21 (1.16-1.26)** | **1.17 (1.12-1.22)** | **1.20 (1.15-1.25)** |
| Second-degree relatives | 2,745,176 | 4,584 (0.17) | 3,922,189 | 5,125 (0.13) | **1.18 (1.13-1.23)** | **1.16 (1.11-1.21)** | **1.17 (1.12-1.23)** |
| Maternal half-siblings | 352,727 | 1,732 (0.49) | 461,335 | 1,802 (0.39) | **1.22 (1.14-1.31)** | **1.20 (1.11-1.28)** | **1.21 (1.13-1.30)** |
| Paternal half-siblings | 400,033 | 1,712 (0.43) | 541,687 | 2,008 (0.37) | **1.10 (1.03-1.18)** | **1.09 (1.02-1.17)** | **1.10 (1.03-1.18)** |
| Aunt/uncle | 1,992,416 | 1,140 (0.06) | 2,919,167 | 1,315 (0.05) | **1.23 (1.12-1.35)** | **1.21 (1.10-1.33)** | **1.22 (1.12-1.34)** |
| Cousins | 6,488,890 | 16,345 (0.25) | 10,651,332 | 23,532 (0.22) | **1.08 (1.06-1.11)** | **1.07 (1.05-1.10)** | **1.08 (1.06-1.11)** |
| ^a^ Model 1 examines the risk of tic disorders for relatives of probands with infections, adjusted for the proband’s and relative’s sex and birth year (categorical).  ^b^ Model 2 is based on Model 1, additionally adjusting for infections in the relatives.  ^c^ Model 3 is based on Model 1, additionally adjusting for tic disorders in the probands.  *Abbreviations:* CI, confidence intervals; OR, odds ratio. | | | | | | | |

**Supplementary Table 3: Proportion of tic disorders in relatives of probands diagnosed with 1, 2, and 3 or more lifetime infections vs in relatives of probands with no infections.**

|  | **Number of infections in the proband** | | |
| --- | --- | --- | --- |
|  | **1 infection** | **2 infections** | **3 or more infections** |
|  | OR (95% CI) | OR (95% CI) | OR (95% CI) |
| Individual^a^ | **1.25 (1.19 - 1.31)** | **1.54 (1.44 - 1.65)** | **1.93 (1.81 - 2.06)** |
| First-degree relatives^b^ | **1.09 (1.04 - 1.14)** | **1.25 (1.17 - 1.34)** | **1.54 (1.43 - 1.65)** |
| Parents^b^ | 1.08 (0.94 - 1.24) | 1.20 (0.99 - 1.46) | **1.65 (1.34 - 2.04)** |
| Full siblings^b^ | **1.09 (1.04 - 1.15)** | **1.27 (1.18 - 1.36)** | **1.53 (1.42 - 1.64)** |
| Second-degree relatives^b^ | **1.10 (1.04 - 1.15)** | **1.26 (1.18 - 1.35)** | **1.33 (1.24 - 1.44)** |
| Maternal half-siblings^b^ | **1.10 (1.01 - 1.19)** | **1.36 (1.22 - 1.52)** | **1.42 (1.27 - 1.59)** |
| Paternal half-siblings^b^ | 1.06 (0.98 - 1.15) | **1.12 (1.00 - 1.24)** | **1.20 (1.07 - 1.36)** |
| Aunt/uncle^b^ | **1.14 (1.04 - 1.26)** | **1.33 (1.16 - 1.53)** | **1.39 (1.18 - 1.64)** |
| Cousins^b^ | **1.05 (1.03 - 1.08)** | **1.10 (1.06 - 1.14)** | **1.16 (1.11 - 1.22)** |
| ^a^ The within-individual analysis was adjusted for sex and birth year (categorical).  ^b^ The analyses in the relatives were adjusted for the proband’s and relative’s sex and birth year (categorical).  *Abbreviations:* CI, confidence intervals. | | | |

**Supplementary Figure 2: Hazard ratio for tic disorders in relatives of probands diagnosed with bacterial infections.**

**
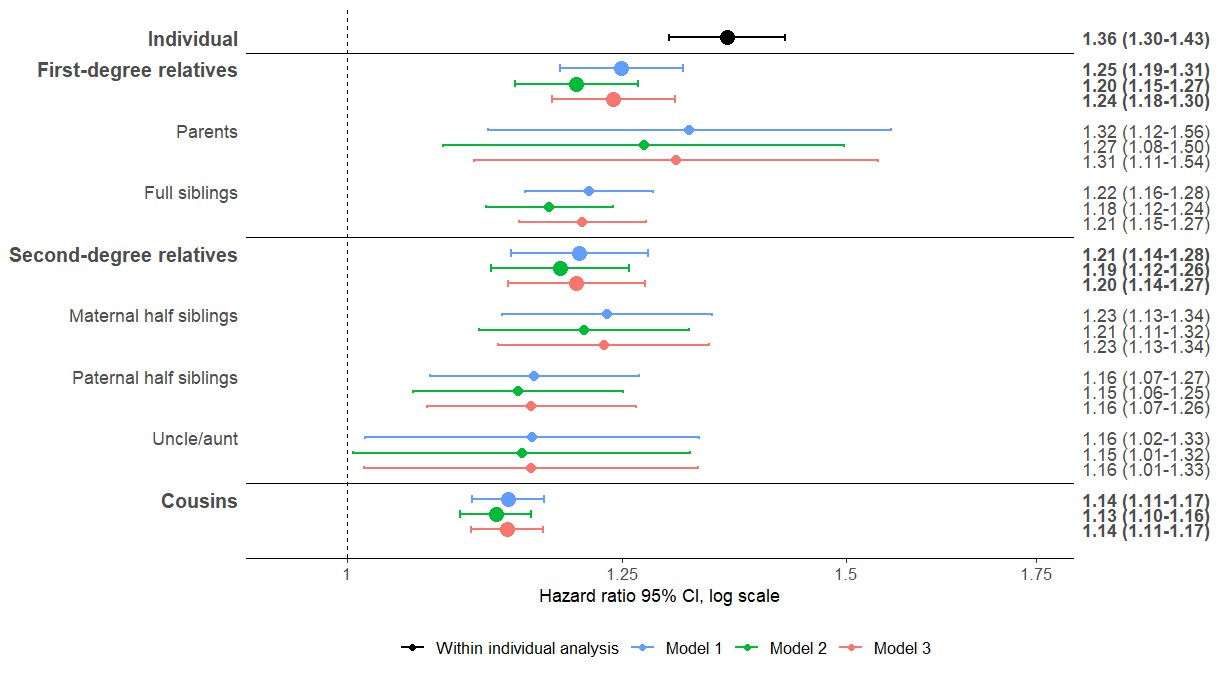
**

**Footnote:**

The within-individual analysis (black) was adjusted for sex and birth year (categorized in 10-year increments).

Model 1 (blue) examines the risk of tic disorders for relatives of probands with bacterial infections, adjusted for the proband’s and relative’s sex and birth year (categorized in 10-year increments).

Model 2 (green) is based on Model 1, additionally adjusting for bacterial infections in the relatives.

Model 3 (red) is based on Model 1, additionally adjusting for tic disorders in the probands.

*Abbreviations:* CI, confidence intervals.

**Supplementary Figure 3: Hazard ratio for tic disorders in relatives of probands diagnosed with viral infections.**

**
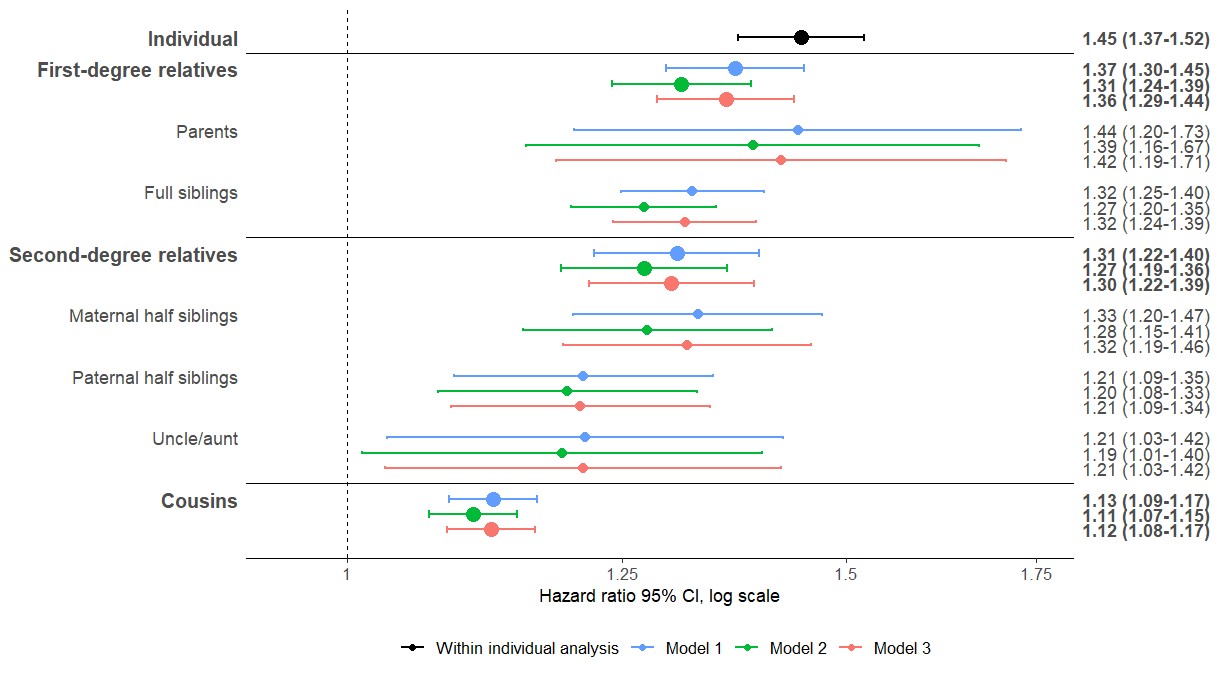
**

**Footnote:**

The within-individual analysis (black) was adjusted for sex and birth year (categorized in 10-year increments).

Model 1 (blue) examines the risk of tic disorders for relatives of probands with viral infections, adjusted for the proband’s and relative’s sex and birth year (categorized in 10-year increments).

Model 2 (green) is based on Model 1, additionally adjusting for viral infections in the relatives.

Model 3 (red) is based on Model 1, additionally adjusting for tic disorders in the probands.

*Abbreviations:* CI, confidence intervals.

Supplementary Table 4: Characteristics of the study cohort for the subgroup analyzed for primary care infections.

|  |  | **Infections**  (N=424,784)  *n (%)* | **No infection**  (N=170,923)  *n (%)* |
| --- | --- | --- | --- |
| Tic disorders | No record of tic disorders | 423,381 (99.7) | 170,569 (99.8) |
|  | Tic disorders | 1,403 (0.3) | 354 (0.2) |
| Sex | Male | 210,017 (49.4) | 95,923 (56.1) |
|  | Female | 214,767 (50.6) | 75,000 (43.9) |
| Birth year | 1980-1987 | 72,198 (17.0) | 59,567 (34.8) |
|  | 1988-1994 | 99,508 (23.4) | 60,001 (35.1) |
|  | 1995-2001 | 108,695 (25.6) | 29,168 (17.1) |
|  | 2002-2008 | 144,383 (34.0) | 22,187 (13.0) |
|  | | | |

Supplementary Table 5: Number of probands in the subgroup analyzed for primary care infections and each cluster of relatives.

| **Total cohort and family clusters** | **Unique probands** | **Unique pairs^a^** | **Observations^b^** | **Excluded** |
| --- | --- | --- | --- | --- |
| Total cohort | 595,707 | NA | 595,707 | NA |
| Full siblings | 424,952 | 306,755 | 613,510 | 170,755^c^ |
| Half-siblings | 107,511 | 94,478 | 188,956 | 488,196^c^ |
| Cousins | 329,247 | 600,303 | 1,200,606 | 266,460^d^ |
| Note: In all pairs of siblings and cousins, each individual contributed to the analysis, at least once, with information on exposure and on outcome.  ^a^ Number of the unique pairs identified (e.g., Offspring- Mother, Sibling1–Sibling2).  ^b^ Number of observations included in the analysis (i.e., all possible combinations of pairs in which members contribute to the analysis with information on exposure and outcome).  ^c^ Probands with no siblings of a certain degree of relatedness identified from the study cohort.  ^d^ Probands with no cousins identified from the study cohort, double cousins, or if parents of cousins are twins. | | | | |

**
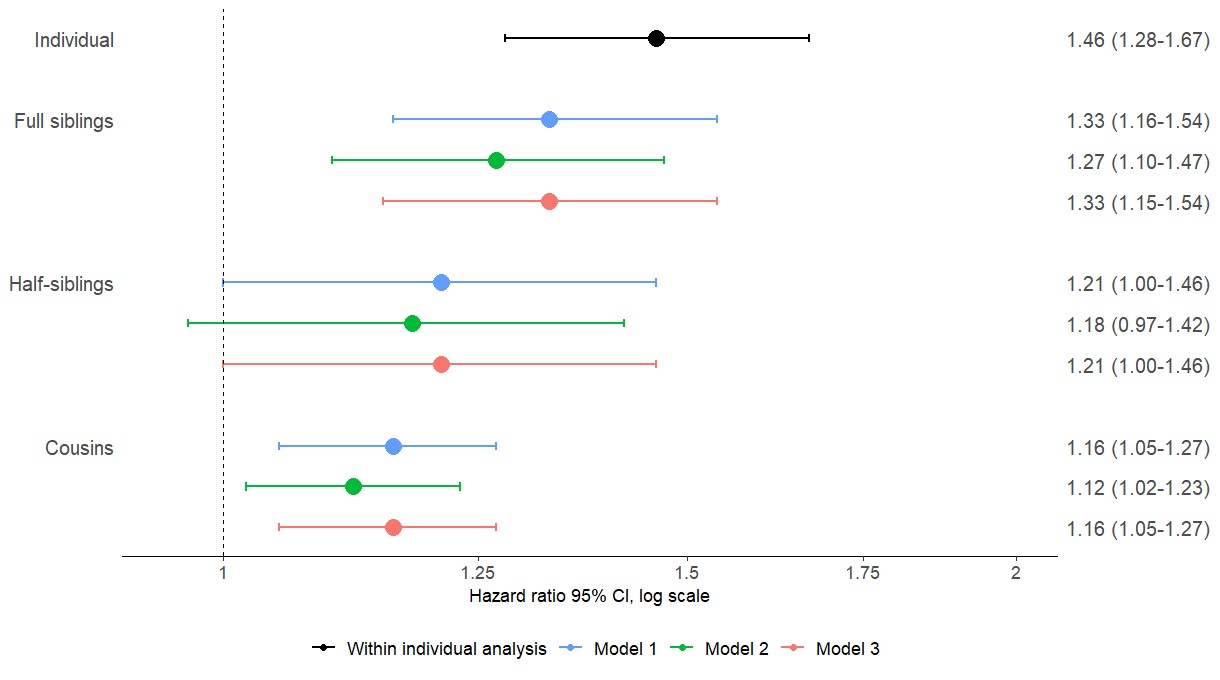
Supplementary Figure 4: Hazard ratio for tic disorders in relatives of probands diagnosed with infections diagnosed in primary care services.**

**Footnote:**

The within-individual analysis (black) was adjusted for sex and birth year (categorized in 7-year increments).

Model 1 (blue) examines the risk of tic disorders for relatives of probands with infections, adjusted for the proband’s and relative’s sex and birth year (categorized in 7-year increments).

Model 2 (green) is based on Model 1, additionally adjusting for infections in the relatives.

Model 3 (red) is based on Model 1, additionally adjusting for tic disorders in the probands.

*Abbreviations:* CI, confidence intervals.
